# Social anxiety symptoms and their relationship with suicidal ideation and depressive symptoms in adolescents: A prospective study

**DOI:** 10.1002/jcv2.12249

**Published:** 2024-06-10

**Authors:** Kenny Chiu, Argyris Stringaris, Eleanor Leigh

**Affiliations:** ^1^ Department of Clinical Psychology and Psychological Therapies University of East Anglia Norwich UK; ^2^ Divisions of Psychiatry and Psychology and Language Sciences University College London London UK; ^3^ First Department of Psychiatry Aiginiteion Hospital National and Kapodistrian University of Athens Athens Greece; ^4^ Department of Experimental Psychology University of Oxford Oxford UK

**Keywords:** adolescent, depression, social anxiety, social phobia, suicide, youth

## Abstract

**Background:**

Social anxiety disorder typically emerges in adolescence and its symptoms often co‐occur with depression and suicidal ideation. It is important to understand whether social anxiety symptoms precede depression and suicidal ideation in youth. This study aimed to investigate the temporal associations between baseline social anxiety and later suicidal ideation and depressive symptoms in a community youth sample.

**Methods:**

The Wellcome Trust NSPN (Neuroscience in Psychiatry Network) study recruited 2397 youth aged 14–24 in the United Kingdom to participate in a prospective observational study. Participants were assessed for symptoms of social anxiety, generalised anxiety, depression and suicidal ideation at baseline, 1‐year follow‐up, and 2‐year follow‐up. We conducted multiple linear regression analyses and mediation analyses to examine whether baseline social anxiety was associated with 2‐year suicidal ideation and depressive symptoms (excluding suicide‐related items), and whether these associations were mediated by 1‐year depressive symptoms. The study was preregistered on the Open Science Framework.

**Results:**

Results from multiple linear regression analyses indicated that baseline social anxiety symptoms were associated with 2‐year suicidal ideation (*β* = 0.07, *p* < 0.05, 95% CI [0.02, 0.12]) and 2‐year depressive symptoms (*β* = 0.08, *p* < 0.05, 95% CI [0.02, 0.13]), after controlling for baseline predicted variable. Furthermore, 1‐year depressive symptoms significantly mediated the relationship between baseline social anxiety symptoms and 2‐year suicidal ideation (*β* = 0.04, 95% CI [0.02, 0.05]), and the relationship between baseline social anxiety symptoms and 2‐year depressive symptoms (*β* = 0.06, 95% CI [0.03, 0.08]) after adjusting for age, sex, and other covariates.

**Conclusions:**

We found evidence that baseline social anxiety symptoms were associated with 2‐year suicidal ideation and 2‐year depressive symptoms via 1‐year depressive symptoms in non‐clinical adolescents. These results may have important implications for targeted psychological interventions.

**Key points**

- Social Anxiety Disorder (SAD) emerges in adolescence and its symptoms often co‐occur with depressive symptoms and suicidal ideation. The temporal relationship between social anxiety, depressive symptoms, and suicidal ideation remains unclear due to limited research.
- In a community youth sample, we found evidence that baseline social anxiety was a significant predictor of 2‐year suicidal ideation and 2‐year depressive symptoms. These relationships were mediated by 1‐year depressive symptoms, after controlling for age, sex and other covariates.
- These findings highlight the importance of assessing suicide risks in adolescents with social anxiety, as well as the potential benefit of addressing social anxiety to alleviate persistent low mood in adolescents.

## INTRODUCTION

Social Anxiety Disorder typically develops in adolescence (Kessler et al., 2005) and is associated with multiple adverse outcomes during adolescence, such as poorer social functioning (Chiu et al., 2021), poorer academic performance (Leigh et al., 2021a), suicidal ideation (Gallagher et al., 2014), and depressive symptoms (Hamilton et al., 2016). Anxiety as a risk factor for suicide has received increased attention over recent years (Bentley et al., 2016; Kanwar et al., 2013; Too et al., 2019). A meta‐analysis has found that anxiety disorder diagnosis is a significant predictor of suicidal ideation (Bentley et al., 2016). The relationship between social anxiety and suicidal risk is relatively less explored. A systematic review and meta‐analysis found evidence of a concurrent association between social anxiety and suicidal ideation in young people (Leigh et al., 2023). However, evidence for a prospective association between social anxiety and suicidal ideation was limited. Using a clinical sample of inpatient adolescents aged 12–15 years recruited in the US, Gallagher et al. (2014) reported evidence of a significant positive correlation between baseline social anxiety and 18‐month suicidal ideation (*r* = 0.32). Furthermore, they found a significant indirect effect from baseline social anxiety symptoms to 18‐month suicidal ideation through loneliness, even after adjusting for sex, number of psychiatric diagnoses, baseline depression symptoms, and baseline suicidal ideation. Their findings suggest social anxiety may be a specific factor that confers vulnerability to suicidality (Joiner, 2007). In another study involving non‐clinical adolescents aged 10–17 years old, Zhu et al. (2021) found that baseline social anxiety symptoms were not significantly associated with the onset of suicidal ideation measured at 9 months, when age, sex, generalised anxiety symptoms, trait anxiety, and other covariates were controlled for. However, this study focussed on the onset of suicidal ideation, not its severity. Given these limited findings, more observational studies are needed to examine the temporal relationship between social anxiety and suicidal ideation in young people.

Anxiety is known to be related to the persistence of depression in adults (Coryell et al., 2012; Gaynes et al., 1999). However, the role of social anxiety in explaining the persistence of depression in adolescence remains less explored. In a clinical sample of youth aged 14–24 years old, Stein et al. (2001) found that individuals with SAD were at an increased risk of developing clinical depression at 3‐ to 4‐year follow‐up. Furthermore, they found that those who experienced SAD and depressive disorder at baseline were more likely to experience persistent depression over time, compared to those with depressive disorder without comorbid SAD. Considering the adverse effects that depression can have on adolescents' long‐term functional outcomes (Clayborne et al., 2019), future research is needed to clarify whether social anxiety symptoms are associated with the persistence of depression over time.

Understanding the temporal relationships of social anxiety with suicidal ideation and depressive symptoms in adolescence may have implications for early recognition, clinical prediction, and psychological interventions. The present study aimed to examine the associations of baseline social anxiety with future suicidal ideation and depressive symptoms, using survey data from a large observational study conducted in the UK. Considering the findings reported by Gallagher et al. (2014), we hypothesised that baseline social anxiety symptoms would explain the variances of 2‐year suicidal ideation even after controlling for baseline suicidal ideation (Hypothesis 1). In addition, we hypothesised that 1‐year depressive symptoms (excluding suicide related items) would have a statistically significant indirect effect in the association between baseline social anxiety and 2‐year suicidal ideation, even after controlling for effects from age, sex, and suicidal ideation, depressive symptoms (excluding suicide related items), and generalised anxiety symptoms (Hypothesis 2). This is because prospective studies have provided evidence of a significant association between social anxiety and later depressive symptoms (Belmans et al., 2019), and a significant association between depression symptoms and suicidal ideation in adolescents (Hill et al., 2018; Zhu & Wong, 2022). We included generalised anxiety symptoms as a covariate because anxiety has been suggested to be a risk factor for suicide ideation (Bentley et al., 2016; Kanwar et al., 2013; Too et al., 2019), and generalised anxiety and social anxiety often co‐occur in young people (Chavira et al., 2004; Walkup et al., 2008). Drawing from literature on the association between SAD and persistence of depression (Stein et al., 2001) and the relatively high prevalence of SAD during adolescence (Kessler et al., 2005), we hypothesised that baseline social anxiety symptoms would be associated with 2‐year depressive symptoms even after controlling for baseline depressive symptoms (Hypothesis 3). Furthermore, we hypothesised that 1‐year depressive symptoms would have a significant indirect effect in the association between baseline social anxiety and 2‐year depressive symptoms, after accounting for the effects of age, sex, depressive symptoms, and generalised anxiety symptoms (Hypothesis 4).

## METHODS

### Participants and procedure

The study data originated from the Wellcome Trust Neuroscience in Psychiatry Network (Kiddle et al., 2018). This research initiative aimed to recruit 2000 healthy volunteers, with equal numbers of males and females for five age groups. Participants between the ages of 14–24 were recruited from 50 GP clinics and schools in Cambridgeshire, London, and surrounding areas between 2012 and 2017. Parental consent was sought for participants who were under the age of 18. Of the 4170 participants who returned an expression of interest form, 3726 of them received a questionnaire pack, 2397 completed the questionnaire pack which was designed to assess participants' mental health and behaviours, including their experiences of depressive symptoms, suicidal ideation, social anxiety symptoms, and generalised anxiety symptoms. The present sample consisted of 2397 participants who reported data at baseline (T0), 1‐year follow‐up (T1), and 2‐year follow‐up (T2).

### Measures

*Social anxiety symptoms*: Social anxiety symptoms were measured with the Revised Children's Manifest Anxiety Scale (RCMAS) (Reynolds & Richmond, 1985). The RCMAS is a 37‐item self‐report measure designed to measure symptoms of anxiety in children and adolescents aged 6–19 years. Confirmatory factor analysis revealed a four‐factor structure, including social evaluation/oversensitivity, worry, anxious arousal, and dysphoric mood/low self‐concept (White & Farrell, 2001). The social evaluation/oversensitivity subscale consists of six items. Example items include “*I worry about what other people think of me*”, “*I feel that others do not like the way I do things*”, “*I worry about what my parents will say to me*”, and “*I feel someone will tell me I do things the wrong way*”. The six items were scored on a 0–3 scale, with a total score of 0–18. The RCMAS social evaluation/oversensitivity subscale has been used as a measure of social anxiety (McClure & Nowicki, 2001; Messenger et al., 2015). Higher scores on the subscale are indicative of higher levels of social anxiety. Cronbach's alphas for the social anxiety subscale at T0–T2 were 0.85, 0.86, and 0.87 respectively.

*Generalised anxiety symptoms*: Generalised anxiety symptoms were assessed with the 8‐item RCMAS worry subscale, with a total score ranges from 0 to 24. Examples include “*I have trouble making up my mind*” and “*I get nervous when things do not go the right way for me*”. The higher the score on the worry subscale, the higher the level of worry. Cronbach's alphas for the worry subscale at T0–T2 were 0.87, 0.89, and 0.90 respectively.

*Depressive symptoms*: The Short Mood and Feelings Questionnaire (SMFQ) (Angold et al., 1995), a 13‐item self‐report questionnaire, was used to assess depressive symptoms in youth. Items include “*I felt miserable or unhappy*” and “*I didn't enjoy anything at all*”. Each item was scored on a 0–2 scale, with a total score ranging from 0 to 26. A higher score indicate more severe level of depressive symptoms. The SMFQ does not have items on suicidal ideation. Cronbach's alphas for the SMFQ at T0–T2 were 0.90, 0.90, and 0.92 respectively.

*Suicidal ideation:* Participants completed the 4‐item subscale from the MFQ (Angold et al., 1995), which has been validated for use (Hammerton et al., 2014). These items are “*I thought of killing myself*”, “*I wanted to die*”, “*I thought I would be better off dead*”, and “*I thought about how I might do it*”. Each item was rated on a 0–2 scale, with a total score ranging from 0 to 8. A higher score indicates more frequent experience of suicidal ideation. Cronbach's alphas for the suicidal ideation subscale at T0–T2 were 0.89, 0.87, and 0.89 respectively.

### Data analysis

Analyses were performed using R Studio (R Core Team, 2020). Data distribution was examined by inspecting histograms. Percentages of missing data were evaluated. Little's missing completely at random (MCAR) test (Little, 1988) was conducted to examine if data was MCAR. In the present sample, baseline suicidal ideation, 1‐year social anxiety symptoms, and 2‐year depressive symptoms had the highest percentages of missing data (54%, 52%, and 47% respectively). Little's MCAR test was significant, *X* ^2^ = 501.01, *df* = 296, *p* < 0.05, suggesting data were not MCAR. Linear regression analyses showed that being a male, having a high level of depressive symptoms at any time point, and a high level of baseline social anxiety symptoms significantly predicted data missingness of 2‐year suicidal ideation and 2‐year depressive symptoms (*ps* < 0.05), suggesting that data could be missing at random. Given the high percentage of missing data and data missingness pattern, we used multiple imputation by chained equations to manage missing data. The *mice* package (van Buuren & Groothuis‐Oudshoorn, 2011) was used to generate imputed datasets. Data convergence was examined by inspecting the means and standard deviation of the imputed variables. Multiple linear regression analyses were conducted to test Hypotheses 1 and 3, whereas mediation analyses were used to examine Hypotheses 2 and 4. The *mediation* (Tingley et al., 2014) package was used to estimate mediation effect, direct effect and total effect, by running 1000 simulations to estimate confidential intervals for these effects. We presented findings derived from the imputed data in the manuscript, while the results obtained from a complete‐case analysis can be found in the Supporting Information. In addition, we repeated the analyses by additionally controlling for autoregressive paths and cross‐lagged paths of social anxiety, suicidal ideation and depressive symptoms, and the results can be found in the Supporting Information.

### Study pre‐registration

The study data analysis plan was preregistered on Open Science Framework (https://osf.io/5t6m4). In the pre‐registration, we planned to test our hypotheses in two samples, one of which was a clinical sample of adolescents who experienced depression. Upon review of the clinical sample, we found that each participant attended clinical visits at different time points and these time points were not predetermined. Given the lack of consistency in data availability across time, we concluded that it would not be possible to test our hypotheses using the clinical sample data. Additionally, we initially intended to perform correlational analyses to test Hypotheses 1 and 3. However, we opted for multiple linear regressions analysis after we published the pre‐registration. This decision was made to explore the degree to which baseline social anxiety symptoms could explain variances in 2‐year suicidal ideation or 2‐year depressive symptoms, while controlling for their respective baseline scores.

## RESULTS

### Descriptive statistics and bivariate correlations

The sample consisted of 2397 participants who attended the baseline visit. 77% (*n* = 1831) and 55% (*n* = 1319) of the original sample returned at the 1‐year and 2‐year follow‐up visit respectively (see Table 1). Ethnic distribution broadly resembled the characteristics of the UK general population (Kiddle et al., 2018). A Pearson correlational analysis was performed on the imputed datasets and the pooled statistics were reported (see Table S1 in Supporting Information). The correlation between baseline social anxiety and 2‐year suicidal ideation was statistically significant, *r*(2397) = 0.24, *p* < 0.01, 95% CI [0.23, 0.26]. Additionally, there was a significant positive correlation between baseline social anxiety and 2‐year depressive symptoms, *r*(2397) = 0.40, *p* < 0.01, 95% CI [0.38, 0.41].

**TABLE 1:** Sample characteristics and descriptive statistics for main variables.

	Baseline	1‐year visit	2‐year visit
Characteristics	*n*	*n*	*n*
Sex			
Female	1285	1025	788
Male	1112	806	531
Ethnicity			
Asian/Asian British	214	160	83
African/Caribbean/Black British	91	73	38
White British	1755	1446	776
Mixed/multiple ethnic groups	136	111	61
Other ethnic groups	36	22	13
Decline to state	8	8	3

	Mean (SD)	Mean (SD)	Mean (SD)
Age	19.04 (3.00)	20.16 (3.10)	21.22 (3.10)
Main variables			
Social anxiety symptoms	2.90 (2.05)	2.57 (2.09)	2.43 (2.11)
Suicidal ideation	0.78 (1.56)	0.70 (1.54)	0.63 (1.44)
Depressive symptoms	7.35 (5.49)	6.76 (5.53)	6.42 (5.48)
Generalised anxiety symptoms	4.06 (2.56)	3.72 (2.69)	3.60 (2.73)

*Note*: Social anxiety symptoms = RCMAS social anxiety subscale; Suicidal ideation = MFQ 4 items on suicidal ideation; Depressive symptoms = SMFQ; Generalised anxiety symptoms = RCMAS worry subscale.

Whether baseline social anxiety symptoms explain variances of 2‐year suicidal ideation (Hypothesis 1) and whether this relationship is mediated by 1‐year depressive symptoms (exclude suicide‐related items) (Hypothesis 2).

**FIGURE 1:**
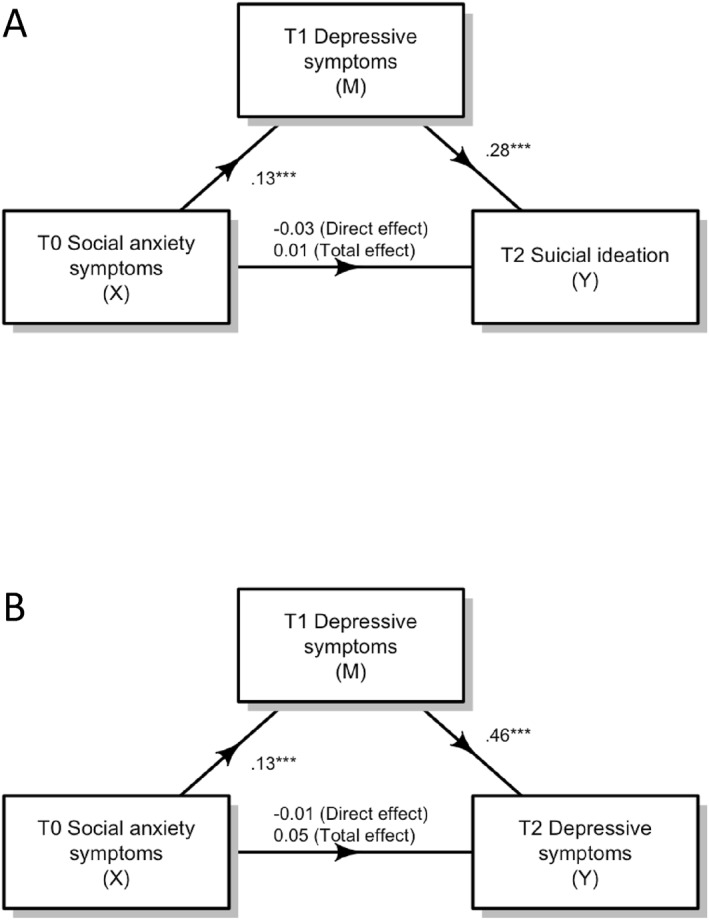
T1 depressive symptoms mediated the relationship between T0 social anxiety symptoms and (A) T2 suicidal ideation and (B) T2 depressive symptoms (controlling for age, sex, and baseline scores). T0 = baseline, T1 = 1‐year follow‐up, T2 = 2‐year follow‐up, X = Predictor variable, M = Mediator, Y = Predicted variable. ***p < 0.001.

Multiple linear regression analysis was conducted to examine if baseline social anxiety was a significant predictor of 2‐year suicidal ideation, while controlling for baseline suicidal ideation. We found that baseline social anxiety significantly explained the variances of 2‐year suicidal ideation. *β* = 0.07, *p* < 0.05, 95% CI [0.02, 0.12]. We examined the mediating role of 1‐year depressive symptoms in this relationship whilst controlling for age, sex, baseline suicidal ideation, baseline generalised anxiety, and baseline depressive symptoms (See Figure 1A). We found a significant indirect effect (*β* = 0.04, 95% CI [0.02, 0.05]), with a non‐significant direct effect (*β* = −0.03, 95% CI [‐0.08, 0.03]) and a non‐significant total effect (*β* = 0.01, 95% CI [‐0.04, 0.06]). The proportion of the total effect mediated was 1.11% (95% CI [‐20.66, 19.59]).

Whether baseline social anxiety symptoms explain variances of 2‐year depressive symptoms (exclude suicide‐related items) (Hypothesis 3), and whether this relationship is mediated by 1‐year depressive symptoms (exclude suicide‐related items) (Hypothesis 4).

Multiple linear regression analysis was conducted to examine if baseline social anxiety was a significant predictor of 2‐year depressive symptoms, while baseline depressive symptoms were controlled for. Results suggested baseline social anxiety significantly explained the variances of 2‐year depressive symptoms, *β* = 0.08, *p* < 0.05, 95% CI [0.02, 0.13]. The mediating role of 1‐year depressive symptoms in this relationship was examined, whilst controlling for age, sex, baseline depressive symptoms, and baseline generalised anxiety symptoms (See Figure 1B). Statistically significant effects were observed for indirect effect (*β* = 0.06, 95% CI [0.03, 0.08]). However, total effect (*β* = 0.05, 95% CI [−0.0004, 0.10]) and direct effect were not significant (*β* = −0.01, 95% CI [−0.05, 0.04]). The proportion of the total effect mediated was 2.08% (95% CI [−0.98, 6.05]).

## DISCUSSION

In this study we examined whether baseline social anxiety symptoms could explain variances of 2‐year suicidal ideation and 2‐year depressive symptoms in a large community youth sample recruited in the UK. We further examined the mediating role of 1‐year depressive symptoms in these associations. We found evidence to support these four hypotheses. Baseline social anxiety was a significant predictor of 2‐year suicidal ideation, even after controlling for baseline suicidal ideation. This finding is consistent with another study that reported a significant positive effect from baseline social anxiety symptoms to 18‐month suicidal ideation (Gallagher et al., 2014). We found a significant correlation between baseline social anxiety and prospective suicidal ideation in this study, which is consistent with the one reported by Gallagher et al. (2014). We observed a small and significant indirect effect from baseline social anxiety to 2‐year suicidal ideation, mediated by 1‐year depressive symptoms, even after controlling age, sex, and other baseline covariates. This finding suggests that depressive symptoms (excluding suicidality items) may serve as one pathway by which social anxiety symptoms increases one's risk of suicidal ideation. For the first time, we found evidence that baseline social anxiety symptoms predicted 2‐year depressive symptoms in a community adolescent sample, and this was mediated through 1‐year depressive symptoms, even after controlling for baseline generalised anxiety symptoms and other covariates. Given that adolescence is characterised by a normative increase in social fear (Haller et al., 2015), social anxiety may play a specific role in the persistence of depressive symptoms in youth.

Social relationships are highly rewarding during adolescence. However, social anxiety may cause adolescents to avoid social situations. Even when they do engage socially, their social anxiety may impact their performance due to the unintended effects of safety behaviours (Leigh et al., 2021b), making them more susceptible to receive negative feedback from peers (Leigh et al., 2021b) or experience peer rejection (Chiu et al., 2021). These negative interpersonal outcomes may trigger a sense of worthlessness (e.g. “*I am a failure*”, “*Nobody wants me around*”) and hopelessness (e.g. “*I will always be alone*”, “*I will never be goodenough for anything or anyone*”), reducing their sense of achievement, connection, and pleasure (Platt et al., 2013), and promoting avoidance. These depressive symptoms can not only maintain their social anxiety, but also lead them to believe that they will never fit in and that they are a burden to others (Arditte et al., 2016). These beliefs around thwarted belongingness and perceived burdensomeness may trigger suicidal ideation (Brailovskaia et al., 2020), as suicide may seem to be the only way (Baumeister, 1990). In addition, these depressive symptoms may persist over years, especially when young people hold negative social fear strongly and avoid social situations.

This study has a number of strengths. Incorporating three‐time‐point data enabled the exploration of mediating mechanisms between social anxiety and suicidal ideation/depressive symptoms. Having a large dataset means that there is sufficient statistical power to test the hypothesised effects. Appropriate missing data management method was applied to account for missing data in a prospective dataset. Nevertheless, this study has several limitations. First, due to the observational nature of the study design, it is not possible to establish evidence of causal relationships. However, the present study method is deemed suitable, as using experimental designs to induce suicidal ideation or depressive mood can be ethically challenging. Second, the social concern and worry subscales from the RCMAS were used to measure social anxiety and generalised anxiety respectively. Although they have face validity, there is limited evidence regarding the psychometric properties of these RCMAS subscales. Third, social anxiety and suicidal ideation were self‐reported, and the strength of correlations between social anxiety and suicidal ideation may have been inflated due to shared method variance.

In terms of clinical implications, our findings tentatively suggest that non‐clinical adolescents who experience social anxiety symptoms may be vulnerable to experience suicidal ideation, and such vulnerability may be related to the presence of depressive mood. Therefore, it may be helpful to evaluate suicidal risks and depressive symptoms comprehensively when supporting socially anxious youth. Furthermore, our findings suggest addressing social anxiety symptoms in adolescence may reduce the persistence of depression, which may, in turn, decrease other long‐term negative outcomes in adulthood (Clayborne et al., 2019).

In sum, in this study we found evidence in a non‐clinical sample that social anxiety symptoms may be prospectively linked to suicidal ideation and depressive symptoms through intermediate depressive symptoms. Therefore, there is value in developing more comprehensive assessment of suicidal risks and depression in socially anxious youth, and offer timely psychological intervention for social anxiety in adolescence.

## AUTHOR CONTRIBUTIONS

**Kenny Chiu:** Conceptualization, Data curation, Formal analysis, Methodology, Software, Visualization, Writing ‐ original draft preparation, Writing ‐ review & editing. **Argyris Stringaris:** Conceptualization, Methodology, Supervision, Writing – review & editing. **Eleanor Leigh:** Conceptualization, Formal analysis, Methodology, Supervision, Writing – review & editing.

## CONFLICT OF INTEREST STATEMENT

The authors have declared that they have no competing or potential conflicts of interest.

### OPEN RESEARCH BADGES

This article has earned a Preregistered Research Designs badge for having a preregistered research design, available at (https://osf.io/5t6m4).

## ETHICAL CONSIDERATIONS

The data used in this study were collected with ethics approval granted by the Cambridge East Research Ethics Committee (reference number 12/EE/0250).

## Supporting information

Supplementary Material S1

## Data Availability

The data that support the findings of this study can be accessed upon request via https://nspn.org.uk.
