# Supplementary material for: Social anxiety symptoms and their relationship with suicidal ideation and depressive symptoms in adolescents: A prospective study
Source: JCPP Adv. 2024 Jun 10;5(1):e12249. doi: 10.1002/jcv2.12249 (PMC11889651; doi:10.1002/jcv2.12249)
Supplement: Supplementary file 1 — Supplementary Material S1 [file JCV2-5-e12249-s001.docx]

**Supporting Information**

Table S1. Correlational matrix of the imputed data (*N* = 2397)

| Variable | *M* | *SD* | 1 | 2 | 3 | 4 | 5 | 6 | 7 | 8 |
| --- | --- | --- | --- | --- | --- | --- | --- | --- | --- | --- |
|  |  |  |  |  |  |  |  |  |  |  |
| 1. T0 Age | 19.04 | 3.00 |  |  |  |  |  |  |  |  |
|  |  |  |  |  |  |  |  |  |  |  |
| 2. T0 Social Anxiety | 2.90 | 2.05 | -.02* |  |  |  |  |  |  |  |
|  |  |  | [-.04, -.00] |  |  |  |  |  |  |  |
|  |  |  |  |  |  |  |  |  |  |  |
| 3. T0 Generalised Anxiety | 4.07 | 2.56 | .09** | .70** |  |  |  |  |  |  |
|  |  |  | [.07, .11] | [.69, .71] |  |  |  |  |  |  |
|  |  |  |  |  |  |  |  |  |  |  |
| 4. T0 Depression | 7.38 | 5.50 | .05** | .68** | .70** |  |  |  |  |  |
|  |  |  | [.03, .06] | [.67, .69] | [.69, .71] |  |  |  |  |  |
|  |  |  |  |  |  |  |  |  |  |  |
| 5. T1 Depression | 6.85 | 5.54 | .02* | .46** | .45** | .57** |  |  |  |  |
|  |  |  | [.00, .04] | [.45, .48] | [.44, .46] | [.56, .59] |  |  |  |  |
|  |  |  |  |  |  |  |  |  |  |  |
| 6. T2 Depression | 6.62 | 5.52 | .03** | .40** | .39** | .54** | .62** |  |  |  |
|  |  |  | [.01, .04] | [.38, .41] | [.38, .41] | [.52, .55] | [.61, .64] |  |  |  |
|  |  |  |  |  |  |  |  |  |  |  |
| 7. T0 Suicidal Ideation | 0.78 | 1.56 | .01 | .43** | .44** | .68** | .45** | .41** |  |  |
|  |  |  | [-.01, .03] | [.42, .44] | [.42, .45] | [.67, .69] | [.44, .47] | [.39, .42] |  |  |
|  |  |  |  |  |  |  |  |  |  |  |
| 8. T1 Suicidal Ideation | 0.71 | 1.56 | .00 | .29** | .29** | .39** | .66** | .40** | .52** |  |
|  |  |  | [-.02, .02] | [.27, .31] | [.28, .31] | [.38, .41] | [.65, .67] | [.39, .42] | [.50, .53] |  |
|  |  |  |  |  |  |  |  |  |  |  |
| 9. T2 Suicidal Ideation | 0.66 | 1.45 | .02* | .24** | .25** | .39** | .43** | .66** | .48** | .48** |
|  |  |  | [.00, .04] | [.23, .26] | [.23, .27] | [.37, .40] | [.42, .45] | [.65, .67] | [.46, .49] | [.46, .49] |
|  |  |  |  |  |  |  |  |  |  |  |

*Note.* *M* and *SD* are used to represent mean and standard deviation, respectively. * indicates *p* < .05. ** indicates *p* < .01.

Table S2. Results of complete case analyses

| Hypothesis 1 | Linear regression analysis was conducted to examine if baseline social anxiety was a significant predictor of 2-year suicidal ideation. While controlling for baseline suicidal ideation, baseline social anxiety significantly predicted 2-year suicidal ideation (*β* = 0.07, *p* < .05, 95% CI [0.01, 0.13]). |
| --- | --- |
| Hypothesis 2 | When examining the mediating role of 1-year depressive symptoms in this relationship, we found a significant indirect effect (*β* = 0.19, 95% CI [0.15, 0.23]), with a significant direct effect (*β* = 0.08, 95% CI [0.02, 0.14]) and a significant total effect (*β* = 0.27, 95% CI [0.21, 0.33]). The proportion of the total effect mediated was 0.71% (95% CI [0.54, 0.92]). |
| Hypothesis 3 | Linear regression analysis was conducted to examine if baseline social anxiety was a significant predictor of 2-year depressive symptoms. While controlling for baseline depressive symptoms, baseline social anxiety did not significantly predict 2-year depressive symptoms (*β* = 0.06, *p* = .09, 95% CI [-0.01, 0.13]). |
| Hypothesis 4 | When examining the mediating role of 1-year depressive symptoms in this relationship, we found a significant indirect effect (*β* = 0.19, 95% CI [0.16, 0.23]), with a significant direct effect (*β* = 0.08, 95% CI [0.01, 0.14]) and a significant total effect (*β* = 0.27, 95% CI [0.21, 0.33]). The proportion of the total effect mediated was 0.71% (95% CI [0.55, 0.94]). |

Table S3. Results of sensitivity analyses: including autoregressive paths and cross-lagged paths

| Hypotheses 2 and 4 | We conducted an exploratory analysis to examine the robustness of the observed indirect effects. Specifically, we wondered if these indirect effects would remain statistically significant after accounting for the consistency in levels of social anxiety, depressive symptoms, and suicidal ideation and their cross-lagged relationships across three time points, after controlling for age, sex, and baseline generalised anxiety symptoms. We ran a structured equation model using the lavaan package (Rosseel, 2012) with 5000 bootstrapping. Our analysis showed that the indirect effect from baseline social anxiety to 2-year suicidal ideation through 1-year depressive symptoms remained statistically significant (*β* = 0.02, *p* < .05, 95% CI [0.001, 0.04]). Similarly, the indirect effect from baseline social anxiety to 2-year depressive symptoms through 1-year depressive symptoms was also statistically significant (*β* = 0.05, *p* < .01, 95% CI [0.01, 0.08]). |
| --- | --- |
